# Phase I–II study of irinotecan (CPT-11) plus nedaplatin (254-S) with recombinant human granulocyte colony-stimulating factor support in patients with advanced or recurrent cervical cancer

**DOI:** 10.1038/sj.bjc.6602076

**Published:** 2004-08-03

**Authors:** H Tsuda, Y Hashiguchi, S Nishimura, M Miyama, S Nakata, N Kawamura, S Negoro

**Affiliations:** 1 Department of Obstetrics and Gynecology, 2-13-22, Miyakojimahondori Miyakojima, Osaka 534-0021, Japan; 2 Department of Clinical Oncology, Osaka City General Hospital, 2-13-22, Miyakojimahondori Miyakojima, Osaka 534-0021, Japan

**Keywords:** combination chemotherapy, adenocarcinoma of uterine cervix, cervical cancer, phase I study, phase II study

## Abstract

Combination chemotherapy with irinotecan (CPT-11) and platinum compounds is effective for treating cervical cancer. Nedaplatin (254-S) is a new cisplatin analogue that achieves a high response rate (53%) in patients with primary cervical cancer. We performed a phase I–II study of combination chemotherapy with CPT-11 plus 254-S for advanced or recurrent cervical cancer. The inclusion criteria were stage IV disease or recurrence. CPT-11 and 254-S were administered intravenously on day 1, while rhG-CSF (50 *μ*g) was given on days 3–12. This regimen was repeated after 4 weeks. Dose escalation was carried out in tandem (CPT-11/254-S: 50/70, 50/80, and 60/80 mg m^−2^). A total of 27 patients (stage IV=seven, recurrence=20) were enrolled. The phase I study enrolled eight patients. At dose levels 1 and 2, no dose-limiting toxicities were observed. At dose level 3, the first two patients developed DLTs. The maximum tolerated dose of CPT-11 and 254-S was 60 and 80 mg m^−2^, respectively, and the recommended doses were 50 and 80 mg m^−2^. Grade 3/4 haematologic toxicity occurred in 67% in phase II study, but there were no grade 3 nonhaematologic toxicities except fot nausea or lethargy. In all 27 patients, there were two complete responses (7%) and 14 Partial responses (52%), for an overall response rate of 59% (95% confidence interval: 39–78%). Among the 12 responders with recurrent disease, the median time to progression and median survival were 161 days (range: 61–711 days) and 415 days (range: 74–801 days). This new regimen is promising for cervical cancer.

Cancer of the uterine cervix is one of the most common malignancies among women and remains the leading female malignancy in developing countries (Thigpen *et al*, 1994). In 1999, about 6500 patients developed cervical cancer in Japan (Sekiya, 2002). In the USA, approximately 13 000 patients developed cervical cancer in 2000 (Robert *et al*, 2001). This tumour is usually radiosensitive and highly curable at an early stage. For patients with stage IV disease or with recurrence after radiotherapy, however, the prognosis is still dismal (Thigpen *et al*, 1995). In such patients, most of the active chemotherapy agents achieve overall response rates of 20–35% when given as monotherapy, with a median response duration of 3–6 months and a survival time of 5–9 months (Thigpen *et al*, 1981; McGuire *et al*, 1996). Many combination chemotherapy regimens have also been explored during the last two decades. High response rates have been obtained in some studies, but it is difficult to assess the relative merits of the various regimens because of differences in patient selection (Buxton *et al*, 1989; Papadimitriou *et al*, 1999).

Nedaplatin (254-S) is a new cisplatin analogue with the same carrier ligands of ammine as cisplatin but has a different leaving group, a five-membered ring structure in which glycolate is bound to the platinum ion as a bidentate ligand (Figure 1

**Figure 1:**
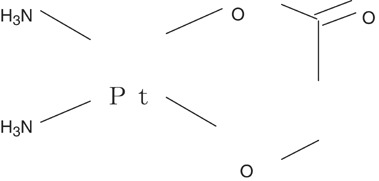
Structure of nedaplatin.

). This product has an approximately 10 times higher water solubility than cisplatin and, unlike cisplatin, shows very limited binding to plasma protein (Sugeno *et al*, 1991). The plasma concentration profile of unbound platinum after 254-S infusion has been reported to be similar to that of total platinum, and the protein binding of 254-S to be lower than that of CDDP (Ota *et al*, 1994). Nedaplatin has a short elimination half-life and a phamacokinetic profile similar to that of CBDCA (Sasaki *et al*, 1989). Nephrotoxicity and gastrointestinal toxicity often limits the clinical use of antitumour agents such as CDDP, but 254-S causes less nephrotoxicity and gastrointestinal toxicity than CDDP, although its haematological toxicity can be a limiting factor at high dosage, as found with CBDCA (Kameyama *et al*, 1990; Ota *et al*, 1992; Suzumura *et al*, 1989). The dose-limiting toxicity (DLT) of 254-S is myelosuppression, especially thrombocytopenia. In the Phase II studies, 254-S monotherapy generated a 46.3% response rate against cervical cancer, especially 53.1% in patients with squamous cell carcinoma (Kato *et al*, 1992).

Irinotecan hydrochloride (CPT-11) is a semisynthetic derivative of camptothecin, an alkaloid contained in plants such as *Camptotheca acuminate* (Nitta *et al*, 1987). Irinotecan inhibits the activity of DNA topoisomerase I, which is necessary for replication of DNA. Several phase II studies have shown that CPT-11 is active against cervical cancer, and activity of CPT-11 monotherapy against recurrent or refractory cervical cancer was revealed in phase II studies performed by the Japan CPT-11 Study Group (24% response rate) and the MD Anderson Cancer Center (21% response rate) (Takeuchi *et al*, 1991; Verschraegen *et al*, 1997). However, a pilot study of CPT-11 in patients with platinum-resistant squamous cell carcinoma failed to show any tumour response (Ivrin *et al*, 1998).

Kanazawa *et al* (2001) reported that the combination of 254-S and CPT-11 showed marked synergistic activity against SBC-3 and PC-14 lung cancer cell lines. This synergistic effect was dependent on the treatment schedule and was produced by concurrent exposure to 254-S and CPT-11. They analysed the mechanism of synergy and demonstrated that the topoisomerase I inhibitory effect of CPT-11 was enhanced 10-fold in the presence of 254-S. Based on these findings, the combination of 254-S and CPT-11 may well be clinically useful. Machida *et al* (2003) performed a phase I study of chemotherapy using CPT-11 plus 254-S for advanced or recurrent cervical cancer. They concluded that the DLT was neutropenia, and their recommended doses of CPT-11 (days 1, 8, and 15) and 254-S (day 1) were 50 and 60 mg m^−2^, respectively. Recombinant human granulocyte colony-stimulating factor (rhG-CSF) can activate haematopoiesis and thus prevent chemotherapy-induced neutropenia or accelerate recovery from this complication, allowing patients to receive full per protocol doses of anticancer drugs. The G-CSF was expected to increase the dose intensity of combination chemotherapy with 254-S plus CPT-11.

Accordingly, we performed a phase I–II study of CPT-11 plus 254-S with rhG-CSF support in patients with advanced or recurrent cervical cancer.

## MATERIALS AND METHODS

### Patient selection

The chief eligibility criteria were as follows: (1) histologically proven cervical cancer (stage IV or recurrent disease), (2) at least one measurable tumour lesion documented radiographically, and (3) an interval >4 weeks between the end of previous treatment (including radiotherapy) and this study. Other eligibility criteria were an age <75 years, performance status (WHO) ⩽2 and life expectancy >3 months. Patients were also required to meet all of the following laboratory criteria: WBC count ⩾3000 mm^−3^ or absolute neutrophil count ⩾1500 mm^−3^, platelet count ⩾100 000 mm^−3^, serum transaminases ⩽60 IU ml^−1^, total bilirubin ⩽1.5 mg dl^−1^, serum creatinine ⩽1.5 mg dl^−1^, and blood urea nitrogen ⩽20 mg dl^−1^. The nature and purpose of the study were fully explained to each patient and all patients gave written informed consent. The study was also approved by the institutional review board of Osaka City General Hospital. Patients were excluded for any of the following conditions: other cancer (metachronous or synchronous); concurrent infection; pre-existing diarrhoea; intestinal paralysis or obstruction; interstitial pneumonia or pulmonary fibrosis; massive ascites; pleural effusion; uncontrolled diabetes; or a history of severe drug hypersensitivity.

### Treatment schedule

A 90-min intravenous infusion of CPT-11 (in 500 ml of 0.9% normal saline) was given on day 1, after which 254-S (in 500 ml of 0.9% normal saline) was also administered intravenously over 90 min. Then, patients received intravenous hydration with 1000 ml of 0.9% saline or 5% dextrose. All patients were treated with a 5-HT3 receptor antagonist before administration of the anticancer drugs. Recombinant human granulocyte colony-stimulating factor (50 *μ*g) was given on days 3–12. Before starting the next cycle, it was confirmed that the leukocyte was ⩾3000 *μ*l^−1^, the neutrophil count was ⩾1500 *μ*l^−1^, and the platelet count was ⩾100 000 *μ*l^−1^, with no diarrhoea, and hepatorenal function meeting the eligibility criteria. Treatment was repeated every 4 weeks for at least two cycles, unless the disease progressed. Treatment was, generally, also stopped if the response was defined as no change (NC) after two cycles. The doses of the two anticancer agents were escalated in tandem, as shown in Table 1

**Table 1:** Dose escalation schedule

**Dose level**	**Irinotecan (mg m^−2^)**	**Nedaplatin (mg m^−2^)**
1	50	70
2	50	80
3	60	80

. Recombinant human granulocyte colony-stimulating factor was also administered when grade 4 neutropenia or grade 3 neutropenia associated with infection occurred. Additionally, if the leukocyte count was <1000 *μ*l^−1^, neutrophil count was <500 *μ*l^−1^, or platelet count was <25 000 *μ*l^−1^ during any cycle, the doses of CPT-11 and 254-S were reduced by one level for the next cycle. Physical examination, complete blood count, and biochemistry tests were carried out weekly.

### Evaluation of response and toxicity

Tumour response was evaluated according to World Health Organization (WHO) criteria (WHO, 1979). Tumours were measured using contrast-enhanced computed tomography (CT) after two cycles of chemotherapy and also 1 month after the end of the treatment. Computed tomography scans were subsequently performed every 3 months for 2 years. The response was assessed from the product of the two largest perpendicular diameters using the following criteria: complete response (CR) was defined as the disappearance of all detectable lesions with no new lesions for at least 4 weeks; partial response (PR) was defined as ⩾50% reduction of the sum of the products of measurable lesions for at least 4 weeks. Progressive disease (PD) was defined as a ⩾25% increase in the sum of the products of all measurable lesions, reappearance of any lesion that had disappeared, or appearance of a new lesion. No change was defined as any outcome that did not qualify as response or progression. Measurements were performed by an experienced radiologist who was blinded to patient information. Patients were considered evaluable for response if they received at least one full cycle of per protocol therapy.

Toxicity was evaluated by the Japan Clinical Oncology Group (JCOG) criteria (Tobina *et al*, 1993). Complete blood counts, biochemistry tests, and liver function tests were performed weekly. Patients were considered evaluable for toxicity if they received at least one full cycle according to the protocol. Dose-limiting toxicity was defined as grade 4 haematologic toxicity (a leukocyte count <1000 *μ*l^−1^, neutrophil count <500 *μ*l^−1^, or platelet count <25 000 *μ*l^−1^), or grades 3–4 nonhaematologic toxicity (except for alopecia, nausea, and vomiting) or failure to recover sufficiently to start the second cycle within 6 weeks. At least three assessable patients were treated at each dose level. If none of these three patients experienced DLT, then the next dose level was started. If one patient developed DLT, the cohort was expanded to six patients. The maximum tolerated dose (MTD) was defined as the dose level at which at least two out of three patients or three out of six patients experienced DLT. The recommended dose (RD) of 254-S and CPT-11 for the subsequent phase II study was set at one level below the MTD.

### Statistical analysis

When the number of subjects required for a 95% confidence interval (95% CI) of ±20% was calculated by setting the expected response rate as 35%, it was 22 subjects. Therefore, the target number of subjects for this study was set as 22. Primary statistical analysis consisted of estimation of the complete and partial response rates. The response rate was calculated as the percentage of complete plus partial responders relative to the total number of assessable patients and 95% CIs for the response were computed using the binomial distribution function.

## RESULTS

### Patient characteristics

A total of 27 patients were enrolled in this study between 10 January 1998 and 1 March 2003. Four patients were in stage IVA, three patients were in stage IVB, and 20 patients had recurrent cancer. Among those recurrent 20 patients, the duration from primary therapy to recurrence was <1 year for 10 patients, from ⩾1 to <2 years for seven patients, and >2 years for three patients. Their median age was 54 years (range: 32–67 years). In all, 22 patients had a PS of 0, four had a PS of 1, and one had a PS of 2. A total of 20 patients had squamous cell carcinoma, four had adenosquamous cell carcinoma, and three had adenocarcinoma. Seven patients had no prior therapy, two had received chemoradiotherapy, five had undergone surgery, and 13 had received both surgery and chemoradiation. The chemoradiotherapy consisted of radiotherapy for whole pelivis and intravenous weekly CDDP treatment (30 mg m^−2^ week^−1^) with or without brachytherapy. The tumour was located in the pelvic cavity in 11 cases, lung in nine cases, liver in five cases, paraaortic lymph nodes in four cases, and Virchow’s node in one case. All patients were assessable for toxicity and response. A total of 71 cycles of therapy were administered. The clinical features of the patients are summarised in Table 2

**Table 2:** Characteristics of the eligible patients (*n*=27)

**Characteristic**	***n***
*Age (years)*	
Median	54
Range	32–67
*WHO PS*	
0	22
1	4
2	1
*FIGO stage*	
IVA	4
IVB	3
Recurrent	20
*Site of recurrent*	
Inside radiation field	5
Outside radiation field	22
*Histology*	
Squamous cell carcinoma	20
Adenosquamous cell carcinoma	4
Adenocarcinoma	3
*Prior therapy*	
None	7
Chemoradiation	2
Surgery	5
Surgery plus chemoradiation	13

.

### Toxicity

#### Phase I study

The phase I study enrolled eight patients. At dose levels 1 and 2, no DLTs were observed. At dose level 1, three patients developed grade 3 neutropenia, while one out of three patients had grade 3 neutropenia at dose level 2. At dose level 2, one out of three patients only received one course because of PD. At dose level three, the first two patients developed grade 4 neutropenia and one of them had febrile neutropenia for 4 days. Both received rhG-CSF and one of them also received intravenous antibiotics. None of the patients experienced nonhaematologic DLTs. In five cases, treatment could be performed every 4 weeks, but treatment delay occurred in two cases (3 days and 7 days). Therefore, the MTD was set as 60 and 80 mg m^−2^ for CPT-11 and 254-S, respectively, and the doses for the phase II study were set at 50 and 80 mg m^−2^. Toxicities are summarised in Tables 3

**Table 3:** Haematologic toxicity

	**Leukopenia**				**Neutropenia**				**Anemia**			**Thrombocytopenia**				
**Dose level**	**G1**	**G2**	**G3**	**G4**	**G1**	**G2**	**G3**	**G4**	**G1**	**G2**	**G3**	**G1**	**G2**	**G3**	**G4**	**Grade 3/4 toxicity (%)**
*Phase I*																
1 (*n*=3)	0	0	3	0	0	1	2	0	0	1	1	1	0	0	0	100
2 (*n*=3)	0	2	1	0	0	2	1	0	1	1	0	0	0	0	0	33
3 (*n*=2)	0	0	1	1	0	0	0	2	0	0	2	0	0	1	1	100
																
*Phase II*																
2 (cycles=50)^a^	6	19	18	1	9	9	22	6	17	17	10	4	8	4	1	62^a^
1 (cycles=8)	0	2	5	1	0	1	3	4	1	2	5	1	2	2	1	100
Total (cycles=58)	6	21	23	2	9	10	25	10	18	19	15	5	10	6	2	67

a Three patients were from the phase I study.

and 4

**Table 4:** Nonhaematologic toxicity

	**Nausea**				**Diarrhoea**				**Haematouria**				**Hepatotoxicity**				**alopecia**		**mucocitis**				**lethargy**			
**Dose level**	**G1**	**G2**	**G3**	**G4**	**G1**	**G2**	**G3**	**G4**	**G1**	**G2**	**G3**	**G4**	**G1**	**G2**	**G3**	**G4**	**G1**	**G2**	**G1**	**G2**	**G3**	**G4**	**G1**	**G2**	**G3**	**G4**
*Phase I*																										
1 (*n*=3)	2	1	0	0	1	0	0	0	0	0	0	0	1	0	0	0	2	1	0	0	0	0	2	1	0	0
2 (*n*=3)	3	0	0	0	1	0	0	0	0	0	0	0	0	0	0	0	2	1	1	0	0	0	3	0	0	0
3 (*n*=2)	1	0	1	0	0	1	0	0	0	1	0	0	0	0	0	0	1	1	1	0	0	0	0	1	1	0
																										
*Phase II*																										
2 (cycles=50)^a^	30	12	4	0	12	7	0	0	5	0	0	0	12	0	0	0	17	8	5	0	0	0	24	7	0	0
1 (cycles=8)	6	2	0	0	3	0	0	0	1	0	0	0	1	0	0	0	2	3	1	0	0	0	8	0	0	0
																										
Total (cycles=58)	36	14	4	0	15	7	0	0	6	0	0	0	13	0	0	0	19	11	6	0	0	0	32	7	0	0

a Three patients were from the phase I study.

.

#### Phase II study

A total of 22 patients, including three patients from the phase I study, were registered for the phase II study. In 7 of 22 patients, grade 4 neutropenia was observed and for these seven patients, the doses of CPT-11 and 254-S were reduced by one level for the next cycle. For phase II study, 50 cycles were administered at dose level 2 and eight cycles were administered at dose level 1. Finally, a total of 58 cycles were administered, with a median of two cycles per person (range: 1–6 cycles). Haematologic toxicities are summarised in Table 3. Grade 3 or 4 leukopenia, grade 3 or 4 neutropenia, grade 3 anemia, and grade 3 or 4 thrombocytopenia occurred in 43% (25 out of 58), 60% (35 out of 58), 26% (15 out of 58), and 14% (8 out of 58) of all cycles, respectively. The seven patients who had grade 4 neutropenia recovered after short-term therapy with rhG-CSF (median: 4 days; range: 3–9 days), and none of them developed febrile neutropenia. The median leukocyte count nadir occurred on day 16 (range: days 12–22). No patient required transfusion, including platelets or red blood cells. Nonhaematologoic toxicities are summarised in Table 4. There were no severe nonhaematologic toxicities. Only two patients received one cycle of chemotherapy because of PD. Treatment delays occurred in 12 patients (median: 7 days; range: 3–12 days). Occurrence of toxicity, including haematologic and nonhaematologic toxicity, did not appear to be associated with the cumulative dose.

### Response

At dose level 1, one out of three patients achieved a clinical response, but there were no responders at dose level 3. In the phase II study (*n*=22), there were two CRs (9%) and 13 PRs (59%), for an overall response rate of 68% (95% CI: 49–84%).

In all 27 patients, there were two CRs (7%) and 14 PRs (52%), for an overall response rate of 59% (95% CI: 39–78%). Complete response occurred in patients with lung and Virchow’s node metastasis as the measurable target lesions. Nine patients had NC (33%) and two patients had PD (7%) (Table 5

**Table 5:** Outcome of treatment

	**Response**				
**Dose level**	**CR**	**PR**	**NC**	**PD**	**Total**
1 (*n*=3)	0	1	2	0	1/3
2 (*n*=22)	2	13	5	2	15/22
3 (*n*=2)	0	0	2	0	0/2
					
Total (*n*=27)	2	14	9	2	16/27

CR=Complete response; PR=Partial response; NC=No change; PD=Progressive disease.

). Among the 12 responders with recurrent disease, the median time to progression and median survival were 161 days (range: 61–711 days) and 415 days (range: 74–801 days). In one CR case, recurrence occurred at 534 days and the patient is now alive with disease at 801 days. Another CR case is now alive without disease at 711 days. In all, 27 cases, the median survival was 394 days (61–801 days).

Table 6

**Table 6:** Response stratified according to various characteristics in all cases

		**Response**				
**Characteristic**	**No. of Patients**	**CR**	**PR**	**NC**	**PD**	**Response rate (%) (95% CI)**
Total	27	2	14	9	2	59.3 (38.8–77.6)
						
*Stage*						
IV	7	0	4	3	0	57.1 (18.4–90.1)
Recurrent	20	2	10	6	2	60.0 (36.1–80.9)
*Histology*						
Squamous cell carcinoma	20	1	11	7	1	60.0 (36.1–80.9)
Nonsquamous cell carcinoma	7	1	3	2	1	57.1 (18.4–90.1)
*Prior therapy*						
No	12	1	7	4	0	66.7 (34.9–90.1)
Yes	15	1	7	5	2	53.3 (26.6–78.7)
*Site*						
Inside radiation field	5	0	2	2	1	40.0 (5.3–85.3)
Outside radiation field	22	2	12	7	1	63.6 (40.7–82.8)
*Age (years)*						
⩽50	10	1	7	1	1	80.0 (44.4–97.5)
>50	17	1	7	8	1	47.1 (23.0–72.2)

shows the responses stratified according to various clinical factors in all cases. The response rate was 57% (4 out of 7) and 60% (12 out of 20) for primary and recurrent cancer, respectively. The response rate was 53% (8 out of 15) and 67% (8 out of 12) for patients with and without prior treatment except for surgery, respectively. Among 22 patients with diseases outside the radiation field, 14 (two CRs and 12 PRs) achieved a clinical response (64%). Among five patients with disease inside the radiation field, two achieved a clinical response (PR: 40%). In the 10 patients less than 50 years old, the response rate was 80%, while it was 47% in the 17 patients more than 50 years old. After chemotherapy, three out of four stage IVA patients received surgery plus chemoradiation and one received chemoradiation alone, and two out of three stage IVB patients received chemoradiotherapy and one received radiotherapy. Among the remaining 20 recurrent patients, one patient received chemoradiation, two patients received radiotherapy, and two had further chemotherapy after CPT-11 plus 254-S. When the response of measurable lesions was analysed, it was seven of 11 (64%) at the primary site, four of nine (44%) for lung, three of five (60%) for liver, and four of five (80%) for lymph nodes.

## DISCUSSION

We conducted a phase I–II study of combination chemotherapy with CPT-11 plus 254-S and rhG-CSF support for advanced or recurrent cervical cancer. At dose level 3 (CPT-11/254-S: 60/80 mg m^−2^), the first two patients developed grade 4 neutropenia and one of them had febrile neutropenia for 4 days. Accordingly, we defined the MTD for CPT-11/254-S as 60/80 mg m^−2^ and the RD for the phase II study as 50/80 mg m^−2^. In the phase II study (*n*=22), 73% of the 22 patients experienced grade 3 or 4 neutropenia, although the seven patients who had grade 4 neutropenia recovered with rhG-CSF support and a good clinical response rate (68%) was achieved. Grade 3 or 4 neutropenia occurred in 60% (35 out of 58) of all cycles in phase II study, respectively. In all 27 patients, there were two CRs (7%) and 14 PRs (52%), for an overall response rate of 59% (95% CI: 39–78%).

Machida *et al* (2003) conducted a phase I study of this therapy for advanced or recurrent cervical cancer and concluded that (1) the DLT was neutropenia, 2) the MTD of CPT-11 (days 1, 8, and 15)/254-S (day 1) was 60/60 mg m^−2^, and (3) the RD was 50/60 mg m^−2^. Their data are concordant with ours. However, Oshita *et al* (2003) performed a phase I–II study in patients with non-small-cell lung cancer and could not find the MTD, while the RD of CPT-11 (days 1 and 8)/254-S was 60/100 mg m^−2^. Their data are somewhat surprising, because a previous study set the RD for 254-S monotherapy at 100 mg m^−2^ (Ota *et al*, 1992). In Oshita's study, 90% of the patients (38 out of 42) had not received prior therapy and 64% (27 out of 42) of the patients were male. In our study and that of Machida, however, 74 and 58% of the patients had received prior therapy and all of the patients were female, so such differences may explain the different results, but further investigation is required.

In previous studies of combination chemotherapy with CPT-11 plus 254-S, CPT-11 was given on days 1 and 8, but we only gave CPT-11 on day 1 in this study for the following reasons: (1) The combination of 254-S and CPT-11 was reported to show marked synergy in SBC-3 and PC-14 lung cancer cell lines (Kanazawa *et al*, 2001), with the synergistic effect being dependent on the treatment schedule and being produced by concurrent exposure to 254-S and CPT-11. They analysed the mechanism of this synergistic effect and demonstrated that the inhibition of topoisomerase I by CPT-11 was enhanced 10-fold in the presence of 254-S. (2) At present, platinum compounds are thought of as key drugs for cervical cancer, so we focused more on the platinum compound in this study based on these findings.

In patients with advanced or recurrent cervical cancer, most active single agents achieve overall response rates of 15–35% (Thigpen *et al*, 1981; Bonomi *et al*, 1985; Takeuchi *et al*, 1991; McGuire *et al*, 1996; Verschraegen *et al*, 1997; Ivrin *et al*, 1998; Morris *et al*, 1998).

Several combination chemotherapy regimens that contain cisplatin have been tested in phase II studies, and objective responses have been documented in 30–70% of the patients, while the median overall survival time ranged between 7 and 12 months (Buxton *et al*, 1989; Murad *et al*, 1994; Long *et al*, 1995; Papadimitriou *et al*, 1997, 1999; Rose *et al*, 1999). Although it is difficult to directly compare the relative merits of the combined regimens with the single agents, combination chemotherapy seems to be superior to single-agent therapy based on these phase II studies. A randomised study performed by the GOG in 438 assessable patients indicated that the combination of cisplatin and ifosfamide achieved a higher response rate and a longer progression-free survival time compared with cisplatin alone. However, the combination was more toxic and there was no difference of overall survival (Omura *et al*, 1997), suggesting the need to develop new combinations for advanced or recurrent cervical cancer. In this study, the overall response rate was 59%, while among the 12 responders with recurrent disease, the median time to progression and median survival time were 161 days (range: 61–711 days) and 415 days (range: 74–801 days), respectively. Thus, the regimen seems to be promising for treating advanced or recurrent cervical cancer.

Brader *et al* (1998) reported that the site of recurrence (inside the radiation field or outside it) and the age of the patient could predict the response to chemotherapy for cervical cancer. In addition, adenocarcinoma is thought to be more resistant to chemotherapy compared with squamous cell carcinoma. In the present study, both squamous cell carcinoma and adenocarcinoma were sensitive to the combination of CPT-11 plus 254-S. However, this regimen tend to be more effective for disease recurring outside the radiation field than for recurrence inside the radiation field (RR; 64 *vs* 40%). In addition, this regimen tend to be more effective for young patients.

In conclusion, the RD of CPT-11/254-S with rhG-CSF was 50/80 mg m^−2^, and this regimen seems to be promising for treating advanced or recurrent cervical cancer.

## References

[bib1] Bonomi P, Blessing JA, Stehman FB (1985) Randomized trial of three cisplatin dose schedules in squamous cell carcinoma of the cervix: a Gynecologic Oncology Group Study. J Clin Oncol 3: 1079–1085 3894589 10.1200/JCO.1985.3.8.1079

[bib2] Brader KR, Morris M, Levenback C, Levy L, Lucas KR, Gershenson DM (1998) Chemotherapy for cervical carcinoma: factors determining response and implications for clinical trial design. J Clin Oncol 16: 1879–1884 9586904 10.1200/JCO.1998.16.5.1879

[bib3] Buxton EJ, Meanwell CA, Hilton C, Mould JJ, Spooner D, Chetiyawardana A, Latief T, Paterson M, Redman CW, Luesley DM (1989) Combination bleomycin, ifosfamide, and cisplatin chemotherapy in cervical cancer. J Natl Cancer Inst 81: 359–361 2464699 10.1093/jnci/81.5.359

[bib4] Ivrin WP, Price FV, Bailey H, Gelder M, Rosenbluth R, Durivage HJ, Potkul RK (1998) A phase II study of irinotecan (CPT-11) for patients with advanced squamous cell carcinoma of the cervix. Cancer 82: 328–333 9445190 10.1002/(sici)1097-0142(19980115)82:2<334::aid-cncr13>3.0.co;2-#

[bib5] Kameyama Y, Okazaki N, Nakagawa M, Koshida H, Nakamura M, Gemba M (1990) Nephrotoxicity of a new platinum compound, evaluated with rat kidney cortical slices. Toxicol Lett 52: 15–24 2356567 10.1016/0378-4274(90)90161-e

[bib6] Kanazawa F, Koizumi F, Koh Y, Nakamura T, Tatsumi Y, Fukumoto H, Saijo N, Yoshida T, Nishio K (2001) *In vitro* synergistic interactions between the cisplatin analogue nedaplatin and the DNA topoisomerase I inhibitor irinotecan and the mechanism of this interaction. Clin Cancer Res 7: 202–209 11205910

[bib7] Kato T, Nishimura H, Yakushiji M, Noda K, Terashima Y, Takeuchi S (1992) Phase II study of 254-S for gynecological cancer. Jpn J Cancer Chemother 19: 695–701 1580643

[bib8] Long III HJ, Cross WG, Wieand HS, Webb MJ, Mailliard JA, Kugler JW, Tschetter LK, Kardinal CG, Ebbert LP, Rayson S (1995) Phase II trial of methotrexate, vinblastine, doxorubicin, and cisplatin in advanced/recurrent carcinoma of the uterine cervix and vagina. Gynecol Oncol 57: 235–239 7729741 10.1006/gyno.1995.1132

[bib9] Machida S, Ohwada M, Fujiwara H, Konno R, Takano M, Kita T, Kikuchi Y, Komiyama S, Mikami M, Suzuki M (2003) Phase I study of combination chemotherapy using irinotecan hydrochloride and nedaplatin for advanced or recurrent cervical cancer. Oncology 65: 102–107 12931014 10.1159/000072333

[bib10] McGuire WP, Blessing JA, Moore D, Lentz SS, Photopulos G (1996) Paclitaxel has moderate activity in squamous cervix cancer: a Gynecologic Oncology Group Study. J Clin Oncol 14: 792–795 8622025 10.1200/JCO.1996.14.3.792

[bib11] Morris M, Brader KR, Levenback C, Burke TW, Atkinson EN, Scott WR, Gershenson DM (1998) Phase II study of vinorelbine in advanced and recurrent squamous cell carcinoma of the cervix. J Clin Oncol 16: 1094–1098 9508195 10.1200/JCO.1998.16.3.1094

[bib12] Murad AM, Triginelli SA, Ribalta JC (1994) Phase II trial of bleomycin, ifosfamide, and carboplatin in metastatic cervical cancer. J Clin Oncol 12: 55–59 7505808 10.1200/JCO.1994.12.1.55

[bib13] Nitta K, Yokokura T, Sawada S, Kunimoto T, Tanaka T, Uehara N, Baba H, Takeuchi M, Miyasaka S, Mudai H (1987) Antitumor activity of novel derivatives of camptothecin. Gan To kagaku Ryoho 14: 850–857 3566296

[bib14] Omura GA, Blessing JA, Vaccarello L, Berman ML, Clarke-Pearson DL, Mutch DG, Anderson B (1997) Randomized trial of cisplatin *versus* cisplatin plus mitolactol versus cisplatin plus ifosfamide in advanced squamous carcinoma of the cervix: a Gynecologic Oncology Group study. J Clin Oncol 15: 165–171 8996138 10.1200/JCO.1997.15.1.165

[bib15] Oshita F, Yamada K, Kato Y, Ikehara M, Noda K, Tanaka G, Nomura I, Suzuki R, Saito H (2003) Phase I/II study of escalating doses of nedaplatin in combination with irinotecan for advanced non-small-cell lung cancer. Cancer Chemother Pharmacol 52: 73–78 12750839 10.1007/s00280-003-0615-y

[bib16] Ota K, Oguma T, Shimamura K (1994) Pharmacokinetics of platinum in cancer patients following intravenous infusion of *cis*-diammine (glycolato) platinum, 254-S. Anticancer Res 14: 1383–1388 8067710

[bib17] Ota K, Wakui A, Mashima N, Niitani H, Inuyama M, Ogawa I, Ariyoshi H, Yoshida O, Taguchi T, Kimura I, Katoh S (1992) Phase II study of a new platinum complex 254-S, *cis*-diammine (glycolato) platinum(II). Jpn J Cancer Chemother 19: 855–861, (in Japanese) 1605663

[bib18] Papadimitriou CA, Dimopoulos MA, Giannakoulis N, Sarris K, Vassilakopoulos G, Akrivos T, Voulgaris Z, Vlahos G, Diakomanolis E, Michalas S (1997) A phase II trial of methotrexate, vinblastine, doxorubicin, and cisplatin in the treatment of metastatic carcinoma of the uterine cervix. Cancer 79: 2391–2395 9191528 10.1002/(sici)1097-0142(19970615)79:12<2391::aid-cncr14>3.0.co;2-m

[bib19] Papadimitriou CA, Sarris K, Moulopoulos LA, Fountzilas G, Anagnostopoulos A, Voulgaris Z, Gika D, Giannakoulis N, Diakomanolis E, Dimopoulos MA (1999) Phase II trial of paclitaxel and cisplatin in metastatic and recurrent carcinoma of the uterine cervix. J Clin Oncol 17: 761–766 10071264 10.1200/JCO.1999.17.3.761

[bib20] Robert TG, Mary BH, Taylor M, Michael T (2001) Cancer statistics 2001. CA Cancer J Clin 51: 15–3611577478

[bib21] Rose PG, Blessing JA, Gershenson DM, McGehee R (1999) Paclitaxel and cisplatin as first-line therapy in recurrent or advanced squamous cell carcinoma of the cervix: a Gynecologic Oncology Group study. J Clin Oncol 17: 2676–2680 10561341 10.1200/JCO.1999.17.9.2676

[bib22] Sasaki Y, Tamura T, Eguchi K, Shinkai T, Fujiwara Y, Fukuda M, Ohe Y, Bungo M, Horichi N, Niimi S, Minato K, Nakagawa K, Saijo N (1989) Pharmacokinetics of (glycolato-*O*,*O*′)-diammine platinum (II), a new platinum derivative, in comparison with cisplatin and carboplatin. Cancer Chemother Pharmacol 23: 243–246 2647312 10.1007/BF00451649

[bib23] Sekiya M (2002) Reports of the gynecologic tumor committee. Acta Obstet Gynaecol Jpn 54: 697–793, (in Japanese)

[bib24] Sugeno K, Mizojiri K, Okabe H, Esumi Y, Takaichi M, Okada Y (1991) Study on the disposition of a new antineoplastic agent, *cis*-diammine (glycolate) platinum(254-S). Iyakuhin Kenkyu 22: 231–242

[bib25] Suzumura Y, Kato T, Ueda R, Ota K (1989) Effect of treatment schedule on antitumor activity of glycolato-*O*,*O*′-diammine platinum(II), a new platinum derivative: comparison with *cis*-diamminedichloroplatinum(II). Anticancer Res 9: 1083–1088 2817790

[bib26] Takeuchi S, Dobashi K, Fujimoto S, Tanaka K, Suzuki M, Terashima Y, Hasumi K, Akiya K, Negishi Y, Tamamaya T, Tanizawa O, Sugawa T, Umesaki N, Sekiba T, Aono T, Nakano H, Noda K, Shiota M, Yakushiji M, Sugiyama T, Hashimoto M, Yamaji A, Takamizawa H, Sonoda T, Takeda Y, Tomoda Y, Ohta M, Ozaki M, Hirabayashi K, Hiura M, Hatae M, Nishigaki K, Taguchi T (1991) A late phase II study of CPT-11 for uterine cervical cancer and ovarian cancer. Gan To kagaku Ryoho 18: 1681–1689 1872624

[bib27] Thigpen T, Shingleton H, Homesley H, Lagasse L, Blessing J (1981) *Cis*-platinum in treatment of advanced or recurrent squamous cell carcinoma of the cervix. A Phase II study of the Gynecologic Oncology Group. Cancer 48: 899–903 7196794 10.1002/1097-0142(19810815)48:4<899::aid-cncr2820480406>3.0.co;2-6

[bib28] Thigpen T, Vance R, Khansur T (1994) Carcinoma of the uterine cervix: current status and future directions. Semin Oncol 21: 43–56 8202720

[bib29] Thigpen T, Vance RB, Khansur T (1995) The platinum compounds and paclitaxel in the management of carcinomas of the endometrium and uterine cervix. Semin Oncol 22, (Suppl 12) 67–75 7481864

[bib30] Tobina K, Kohno A, Shimada Y, Watanabe T, Tamura T, Takeyama K, Narabayashi M, Fukutomi T, Kondo H, Shimoyama M, Suemasu K, members of the Clinical Trial Review Committee of the Japan Clinical Oncology Group (1993) Toxicity grading criteria of the Japan Clinical Oncology Group. Jpn J Clin Oncol 23: 250–257 8411739

[bib31] Verschraegen CF, Levy T, Kudelka AP, Lierena E, Freedman RS, Edwards CL, Hord M, Steger M, Kaplan AL, Kieback D, Fishman A, Kavanagh JJ (1997) Phase II study of irinotecan in prior chemotherapy-treated squamous cell carcinoma of the cervix. J Clin Oncol 15: 625–631 9053486 10.1200/JCO.1997.15.2.625

[bib32] World Health Organization (1979) Handbook for Reporting Results of Cancer Treatment, Offset publication no. 48. Geneva: World Health Organization

